# Side by side comparison of NOTA and DOTA for conjugation efficiency, gallium-68 labeling, and in vivo biodistribution of anti-mesothelin sdAb A1-His

**DOI:** 10.1186/s41181-025-00380-5

**Published:** 2025-08-20

**Authors:** Émilien N’Guessan, Sandrine Bacot, Florian Raes, Julien Leenhardt, Thibault Guenard, Laurent Dumas, Catherine Ghezzi, Daniel Fagret, Charlotte Lombardi, Alexis Broisat, Mitra Ahmadi

**Affiliations:** 1 Université Grenoble Alpes, INSERM U1039, LRB, Grenoble, France; 2 Department of Nuclear Medicine, Université Grenoble Alpes, CHU Grenoble Alpes, Grenoble, France; 3 Université Grenoble Alpes, CNRS U5525, TIMC-TREE, La Tronche, France

**Keywords:** DOTA, NOTA, Gallium-68, Radiolabeling, Theranostics, sdAb, Nanobody, Radiopharmaceuticals, Mesothelin

## Abstract

**Background:**

Mesothelin is a glycoprotein overexpressed in various cancers, with limited expression in healthy tissues. The single-domain antibody (sdAb, or nanobody) A1-His has previously successfully been validated in mice for the SPECT imaging of mesothelin positive tumors following radiolabeling with ^99m^Tc. Our objective was to radiolabel this sdAb with ^68^Ga for PET imaging, exhibiting superior sensitivity and resolution than SPECT in clinical practice. To this aim, it was conjugated to NOTA chelator that is commonly employed for ^68^Ga labeling of antibody-derived tracers. In addition, the high affinity and specificity of A1-His sdAb position it as a promising candidate for theranostic applications. In anticipation of future radiolabeling with beta-emitting radionuclides, DOTA-conjugated A1-His was also evaluated. Given the high thermal stability of sdAbs, this DOTA-conjugated sdAb could potentially be implemented in future studies as a theranostic agent with beta-emitting radionuclides.

**Results:**

A1-His was successfully conjugated to p-SCN-Bn-DOTA and p-SCN-Bn-NOTA under optimized conditions, achieving chelator-to-sdAb ratios of 1.8 and 1.3, respectively. NOTA-A1-His allowed rapid radiolabeling with ^68^Ga at room temperature, achieving high radiochemical purity (> 98%) within 5 min. Using DOTA, similar purity was obtained at 60 °C for 15 min. Both radiotracers demonstrated stability over 4 h in the radiolabeling medium and 2 h in human blood. However, some instability was observed in murine blood. Biodistribution and imaging studies in mice bearing mesothelin-expressing tumors showed specific tumor targeting for both tracers. Notably, [68Ga]Ga-DOTA-A1-His exhibited twofold lower kidney uptake compared to [68Ga]Ga-NOTA-A1-His, potentially enhancing imaging contrast and reducing renal radiation exposure. His-tag removal, further improves the biodistribution profile of the 2 tracers.

**Conclusions:**

Both p-SCN-Bn-DOTA and p-SCN-Bn-NOTA chelators can be effectively conjugated to the A1 sdAb and radiolabeled with ^68^Ga, producing stable radiotracers with specific tumor-targeting capabilities. NOTA chelator offers advantages in rapid, room-temperature radiolabeling. However, DOTA would offer the advantage to be employed for theranostic approaches using β^−^ emitters such as ^177^Lu or ^161^Tb. The lower kidney retention of DOTA-A1 also suggests that its dosimetry, a key factor in theranostic, would be more favorable.

**Supplementary Information:**

The online version contains supplementary material available at 10.1186/s41181-025-00380-5.

## Background

Among the targets of cancer recently identified, the glycoprotein mesothelin has been proposed as a relevant target for tumor diagnosis and therapy (Faust et al. 2022; Montemagno et al. 2020). Our laboratory has developed and fully characterized the single domain antibody (sdAb) [99mTc]Tc-A1-His that targets both murine and human mesothelin. sdAbs, also known as VHHs or nanobodies, constitute the single variable domain of heavy-chain only antibodies found in camelids. They exhibit pharmacokinetics that are extremely favorable for the development of radiopharmaceuticals, including high affinity and specificity combined to a low molecular weight allowing high tumor uptake and fast blood clearance (Cong et al. 2024). In vitro, A1-His binds to recombinant human mesothelin and to mesothelin expressing cells with nanomolar affinity. In vivo in mice bearing human pancreatic ductal adenocarcinoma (PDAC) or triple negative breast cancer (TNBC), mesothelin expression is observable using [99mTc]Tc-A1-His by Single Photon Emission Computed Tomography (SPECT) imaging, while no signal is identifiable in mesothelin negative tumors. The in vivo specificity of [99mTc]Tc-A1-His binding to mesothelin positive tumors was further confirmed by in vivo competition experiments (Montemagno et al. 2019, 2018). [99mTc]Tc-A1-His could therefore be translated to the clinic and be employed as a companion marker of the anti-mesothelin therapies currently undergoing clinical trials such as antibody–drug conjugates like DMOT4039A and BMS-986148 (Weekes et al. 2016; Rottey et al. 2022) or the numerous CART-T Cells currently under development (Zhai et al. 2023). However, for the detection of tumor lesions in the clinical practice including metastasis, Positron Emission Tomography (PET) is usually considered as more relevant than SPECT, due to its higher sensitivity and resolution. Therefore, the goal of this study was to develop and characterize a PET version of A1-His labeled with ^68^Ga.

To this aim, a chelator with rapid kinetics seemed more suitable. However, while fast radiometal incorporation and low energetic barriers for radiometal-chelate complexation are beneficial, they can also result in fast demetallation and low energetic barriers for radiometal dissociation. Achieving the optimal PET agent requires a careful balance, necessitating the investigation of different chelator agents with distinct properties. Various chelators have been developed for trivalent isotopes, each of them offering different levels of thermodynamic and kinetic stability (Price and Orvig 2014; Fersing et al. 2022; Holik et al. 2022; D’Onofrio et al. 2021). Historically, acyclic DTPA-based ligands were considered as a standard in nuclear medicine for radiolabeling (Liu 2008). However, advancements have led to the adoption of macrocyclic ligands such as DOTA, NOTA, and TRAP for ^68^Ga, which provide enhanced stability. NOTA, a hexadentate chelator, is one of the oldest and most successful chelators for ^68^Ga. It is widely regarded as the “gold standard” for Ga^3+^ chelation, allowing rapid radiolabeling at room temperature and excellent in vivo stability. One straightforward solution with proteins is to perform random NOTA chelator coupling on side chain lysins. This has been previously performed by others in preclinical and clinical studies using antibodies or antibody fragments, including sdAb such as [68Ga]Ga-NOTA-anti-HER2 currently in phase II clinical trial (Gondry et al. 2024).

Another possible strategy is to use DOTA chelator. DOTA offers several advantages including an exceptional in vivo stability, a wide range of bifunctional DOTA derivatives and vector conjugates and, owing to its octadentate property, the possibility of complexing both imaging and therapeutic radiometals within the same framework. However, DOTA requires elevated temperatures for radiolabeling with most radiometals, which can be limiting for protein-based tracers such as antibodies or antibody fragments. Interestingly, sdAbs usually exhibit good thermal stability in comparison to other antibody formats. Therefore, DOTA was also considered as an option of PET imaging with ^68^Ga in this study. One great advantage, for future studies, is the possibility of radiolabeling DOTA using beta- emitters, namely ^177^Lu and ^161^Tb, enabling theranostic evaluations and applications.

Several studies have already been conducted to assess the differences between DOTA and NOTA chelators, highlighting the respective advantages and disadvantages of each when radiolabeling vectors with ^68^Ga (Notni et al. 2012; Ray Banerjee et al. 2016). The objectives of this study were to identify the optimal parameters for NOTA and DOTA conjugation on A1-His and for their subsequent radiolabeling with ^68^Ga. Then, their stability was evaluated in human and murine blood and their biodistribution, with or without His-tag, were investigated in mice bearing tumors using PET imaging and gamma-well counting.

## Methods

### SdAbs production

A1-his was cloned into the pet15b expression vector, incorporating a hexahistidine tail. Transformation into SHuffle T7 Express Competent *E. coli* C3029J (New England Biolab) was performed. SdAbs expression was induced with 1 mM isopropyl-β-d-thiogalactoside (IPTG) at 37 °C overnight. Soluble extracts containing sdAbs were obtained through lysozyme and sonication, followed by a centrifugation step. Further purification involved immobilized metal affinity chromatography (IMAC) on Ni–NTA resin (Sigma-Aldrich) and gel filtration on Superdex 75 HR 16/60 (Cytiva) in Tris/NaCl pH 7.4. The affinity of A1-His for human mesothelin was evaluated using surface plasmonic resonance (SPR) as previously described (N’Guessan et al. 2025). To produce sdAbs without the His-tag use in the biodistribution study, the proteins underwent TEV protease cleavage, as previously described (N’Guessan et al. 2025).

### NOTA and DOTA conjugation to A1-His

The derivatives p-SCN-Bn-DOTA and p-SCN-Bn-NOTA were used for DOTA and NOTA coupling, respectively. To achieve the best conjugation rate of NOTA or DOTA to the sdAb, several conditions were tested, closely monitoring the influence of changes in the excess of chelator and the pH on the conjugation rate. A1-His, initially produced in Tris-NaCl pH 7.4, was buffer-exchanged into 0.5 M sodium carbonate buffer, pH 9.0 (sodium carbonate anhydrous–sodium hydrogen carbonate; Sigma-Aldrich) at the desired pH, using PD-10 size exclusion disposable columns (GE Healthcare). A twenty- or 50-fold molar excess of p-SCN-Bn-NOTA (2.5 mg/mL) or p-SCN-Bn-DOTA (3 mg/mL) was added to the A1-His solution (1.5 mg/mL) and the pH was adjusted to 8 or to 9. Each sample was then incubated for 3 h at 25 °C under stirring.

Purifications were performed as described in our latest article (N’Guessan et al. 2025). Briefly, after each conjugation reaction, the pH was adjusted to 7–7.5, and the solution was purified by size exclusion chromatography (SEC) using semi-preparative high-pressure liquid chromatography (HPLC; Shimadzu). The purified conjugates were then concentrated to 1 mg/mL using Amicon Ultra-15 filters (Ultracel® 3 K) for stability purpose. All samples were analyzed by MALDI-TOF MS using an Autoflex Speed mass spectrometer (Bruker Daltonics). After purification and analysis, each DOTA-conjugated sdAb sample was stored at − 80 °C. All samples were analyzed by Maldi-TOF–MS at various time points up to 2 years.

### Gallium-68 labeling parameters

The factors affecting radiolabeling efficiency were explored by examining various parameters, including (a) reaction concentration, (b) pH of radiolabeling reaction and (c) incubation time, for both [68Ga]Ga-DOTA-A1-His and [68Ga]Ga-NOTA-A1-His. The mixture was heated to 60 °C (thermal stability of A1-His) in the presence of DOTA and maintained at room temperature (25 °C) with NOTA. The influence of all these parameters was evaluated by measuring the radiochemical purity (RCP) of each solution through radio-HPLC analysis. Each parameter was tested in triplicate.

Radiochemical purity (RCP), was assessed using reversed-phase high-performance liquid chromatography (RP-HPLC). The analytical HPLC system (Shimadzu) was equipped with a radiodetector (Elysia-Raytest) and a UV detector (Shimadzu), utilizing a C4 reverse-phase column (Symmetry300™, 5 μm, 150 × 4.6 mm; Waters). The elution was performed with solvents A (0.1% trifluoroacetic acid (TFA) in water) and B (0.1% TFA in acetonitrile) at a flow rate of 1 mL/min under the following gradient conditions: 0–1 min, 5% B; 1–5 min, linear gradient from 5 to 90% B; 5–7 min, 90% B; 7–10 min, returning to initial conditions; and 10–12 min, 5% B. The radiochemical purity of each radiolabeled sdAb was determined by integrating the radioactivity peaks corresponding to the sdAb and any free radionuclide or impurities.

- Reaction concentration
  Either 50 µg or 100 µg of each conjugated A1-His (3.5 or 7 nmoles) were added to a mixture containing 1 M ammonium acetate buffer and Ga-68 eluate (145–222 MBq) (IRE ELiT®, Belgium). The pH of the mixture was adjusted to 3–4 and then incubated for 15 min with stirring at 300 rpm, at 60 °C for DOTA-A1-His and at room temperature for NOTA-A1-His.
- pH
  100 µg of DOTA- or NOTA-conjugated A1-His (7 nmoles) in 1 M ammonium acetate were added to Ga-68 eluate (145–222 MBq). The reaction mixtures, adjusted to final pH values of 3, 4 or 5, were incubated for 15 min under stirring at 300 rpm/min, at room temperature for NOTA-A1-His and at 60 °C for DOTA-A1-His.
- Incubation time
  100 µg of DOTA- or NOTA-conjugated A1-His (7 nmoles) in 1 M ammonium acetate were added to Ga-68 eluate (145–222 MBq). The reaction mixtures were adjusted to final pH values of 3 for DOTA- and 4 for NOTA. All samples were incubated for 15 min under stirring at 300 rpm/min, at room temperature for NOTA-A1-His and at 60 °C for DOTA-A1-His. A sample of each reaction medium was withdrawn at 5 min and 10 min, and analyzed by radio-HPLC.

The optimized labeling conditions described above were used to radiolabel the molecules for in vivo experiments. Radiolabeled sdAbs were purified by gel filtration using NAP-5 columns (Sephadex™ G-25 DNA Grade Gel, GE Healthcare) pre-equilibrated with PBS (Sigma-Aldrich, Cat. #D8662), and subsequently sterilized through 0.22 µm filters (Millex®, Millipore) prior to injection into animals.

### Radiolabeling stability

The stability of [68Ga]Ga-DOTA-A1-His and [68Ga]Ga-NOTA-A1-His in the radiolabeled medium at room temperature was evaluated at 2 and 4 h by determining the RCP through radio-HPLC analysis, using the same conditions as immediately after radiolabeling. The experiments were carried out in triplicate. The stability was also evaluated in whole human and murine blood. Each radiotracer was at a concentration of 37 MBq/mL in blood and kept at 37 °C for 120 min. Samples of 150–200 µL were taken at 30, 60, and 120 min, and centrifuged at 4000 g for 2 min to separate plasma from blood cells. Plasma was then treated with 10% TCA to precipitate large proteins. After a second centrifugation at 13,000 g for 3 min, the RCP of supernatant (protein-free plasma fraction) was analyzed by radio-HPLC. Each experiment was conducted in triplicate.

### Hydrophilicity

The partition coefficient (log P), an indicator of hydrophilicity, was determined for each radiolabeled compound by measuring its distribution between 1-octanol and phosphate-buffered saline (PBS). [68Ga]Ga-DOTA-A1-His or [68Ga]Ga-NOTA-A1-His was mixed with PBS and 1-octanol to achieve a 1:1 ratio between PBS and 1-octanol. After vortexing for 1 min, the vial was centrifuged for 3 min at 13,000 rpm to ensure complete separation of the layers. The 1-octanol phase and the buffer phase were then harvested and the radioactivity determined using a dose calibrator. This process was repeated with fresh phosphate buffer and 1-octanol. Log P was calculated as the logarithm of the ratio of radioactivity in the 1-octanol phase to that in the PBS buffer: Log P = log (total counts in 1-octanol / total counts in PBS buffer).

### Cell line and culture conditions

The HCC70 cell line, obtained from the American Type Culture Collection, was cultured in RPMI 1640 medium (Sigma-Aldrich) supplemented with 10% fetal bovine serum (FBS; Sigma-Aldrich) and 1% penicillin–streptomycin (Sigma-Aldrich). Cells were incubated at 37 °C with 5% CO₂.

### Animal and tumor model

Approval for all procedures was obtained from the animal care and ethics committee of Grenoble Alpes University and the ad hoc French minister (APAFIS#19480–2019022616164184 v4). Mice had access to rodent chow and water ad libitum. They were on a 12 h light/dark cycle, temperature was maintained between 20 and 24 °C, and a relative humidity of 40–60%. A one-week acclimatization period was respected prior implantation.

Twenty-seven female Athymic Nudes mice (Janvier, France) at 5–weeks of age were subcutaneously xenografted in the left posterior upper leg with HCC70 cells (3.5 × 10^6^) in 1:1 (v/v) PBS/Matrigel (Corning) mixture. Tumor growth was monitored using a caliper 2 to 3 times a week, allowing tumors to grow for 3–4 weeks to reach approximately 75–150 mm^3^.

### PET-CT imaging

PET/CT acquisitions were performed 1 h after intravenous injection via the tail vein of [68Ga]Ga-NOTA-A1-His, [68Ga]Ga-DOTA-A1-His, [68Ga]Ga-NOTA-A1 or [68Ga]Ga-DOTA-A1. Randomization ensured similar weights and tumor volumes between the two groups (Supplementary Table 1). An intravenous injection of 100 µl of Gelofusin® 4% was performed 1–2 min before the radiotracer injection to reduce the kidney retention. PET/CT acquisitions were performed using two dedicated system (nanoSPECT/CT and nanoPET/MRI; Mediso). Images were quantified after correction for decay and normalization to the injected dose using VivoQuant Software.

### Biodistribution studies

Two hours after the injection and immediately after the image acquisition, the anesthetized mice were euthanized by cervical dislocation and tumors were harvested along with other organs. Tissues were weighed, and tracer activity was determined using a Wizard^2^ γ-counter (Perkin Elmer). The results were corrected for decay, injected dose (ID), and organ weight and were expressed as %ID/g. Tumor-to-liver (T/L), tumor-to-kidney (T/K) and tumor-to-blood (T/B) activity ratios were computed**.**

### Statistical analysis

Mean values ± standard deviation (SD) were compared using nonparametric, two tailed and unpaired tests, i.e. the Mann–Whitney U test for comparison of two groups. P values of 0.05 or less were considered significant. Analysis were conducted with GraphPad 8.0.2 (Prism).

## Results

### NOTA and DOTA conjugation to A1-His

**Fig. 1:**
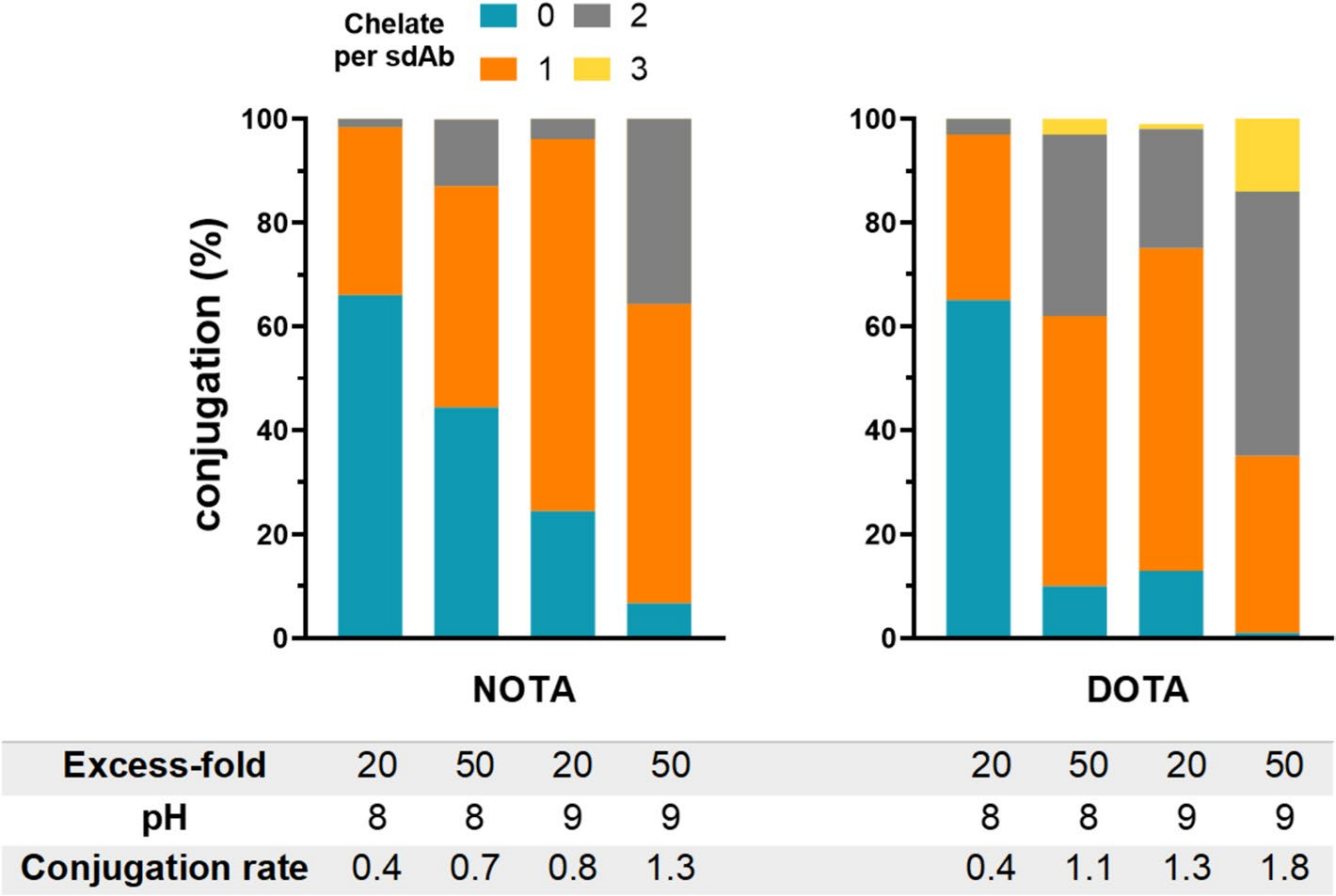
Evaluation of the effect of molar-excess of chelator per sdAb and pH on the conjugation rate

A1-His was successfully produced at a yield of 11.9 mg per liter of Lysogeny Broth culture medium. Similar conjugation rates were observed with NOTA and DOTA using a 20-fold molar excess of chelator at pH 8. Under these conditions, most sdAb remained unconjugated (66% for NOTA and 65% for DOTA), while mono-conjugated sdAb represented 32% for both chelators and bi-conjugated 2 and 3%, respectively. Consequently, the mean conjugation rate was of 0.4 for both chelators. Increasing the excess of chelator to 50 enhanced the conjugation rate to 0.7 for NOTA and 1.1 for DOTA. Similarly, increasing the pH to 9 enhanced the conjugation rate to 0.8 for NOTA and 1.3 for DOTA. These two effects were found to be additive, so that a 50-fold excess of chelator at pH 9 resulted in conjugation rates of 1.3 for NOTA and 1.8 for DOTA (Fig. 1). The 50-fold excess and pH9 were selected as the most potent conditions. Upon storage at − 80 °C at 1 mg/mL, the conjugation remained stable for two years.

### Gallium-68 labeling

Prior to all radiolabeling, the thermal stability of A1-His was assessed using Circular Dichroism analysis, identifying 60 °C as the maximum temperature at which no changes in the secondary structure of A1-His were observed (N’Guessan et al. 2025). This indicates that DOTA radiolabeling can only be performed at temperatures below 60 °C. In contrast, NOTA did not require heating, so all experiments with NOTA were conducted at room temperature (25 °C).

**Fig. 2:**
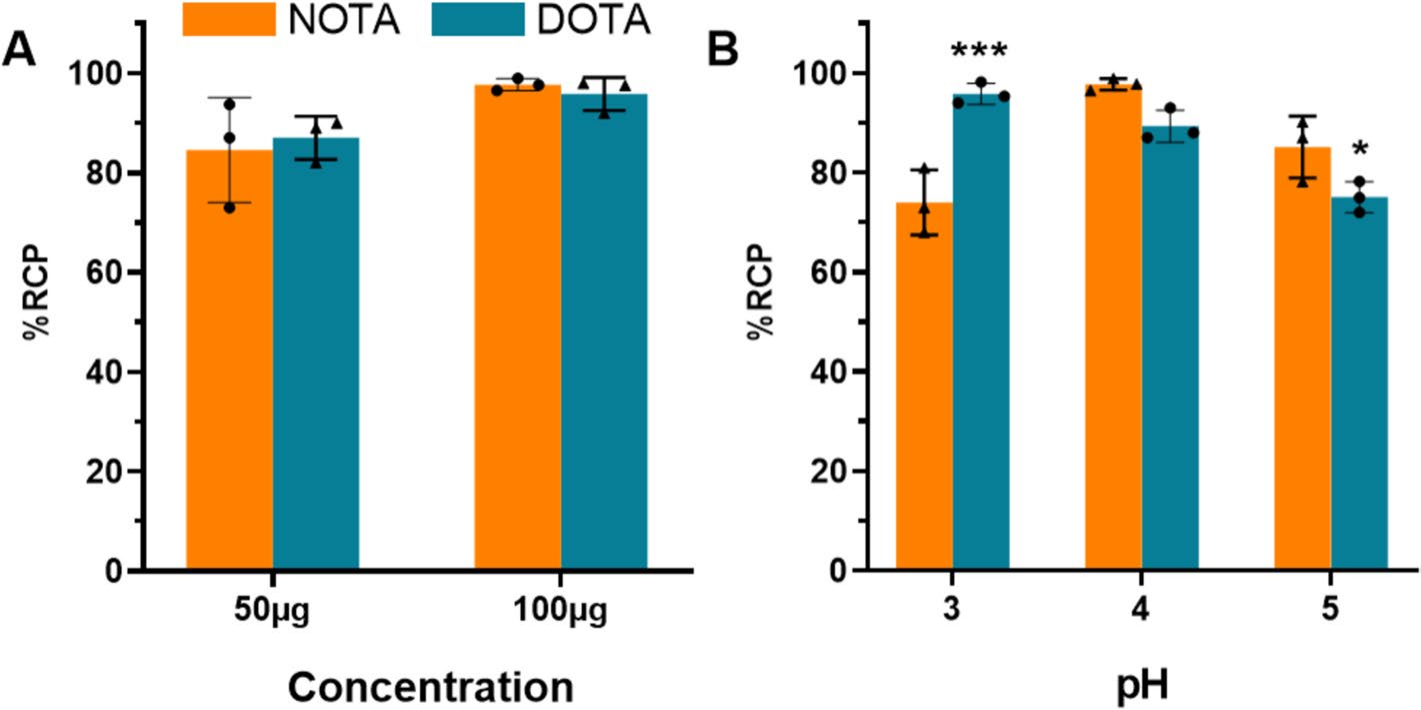
Radiochemical purity of [^68^Ga]Ga-NOTA-A1-His and [^68^Ga]Ga-DOTA-A1-His immediately after radiolabeling as a function of A1-His concentration (**A**) and pH (**B**). *P < 0.05 vs. NOTA and ***P < 0.001 *vs* NOTA

**Fig. 3:**
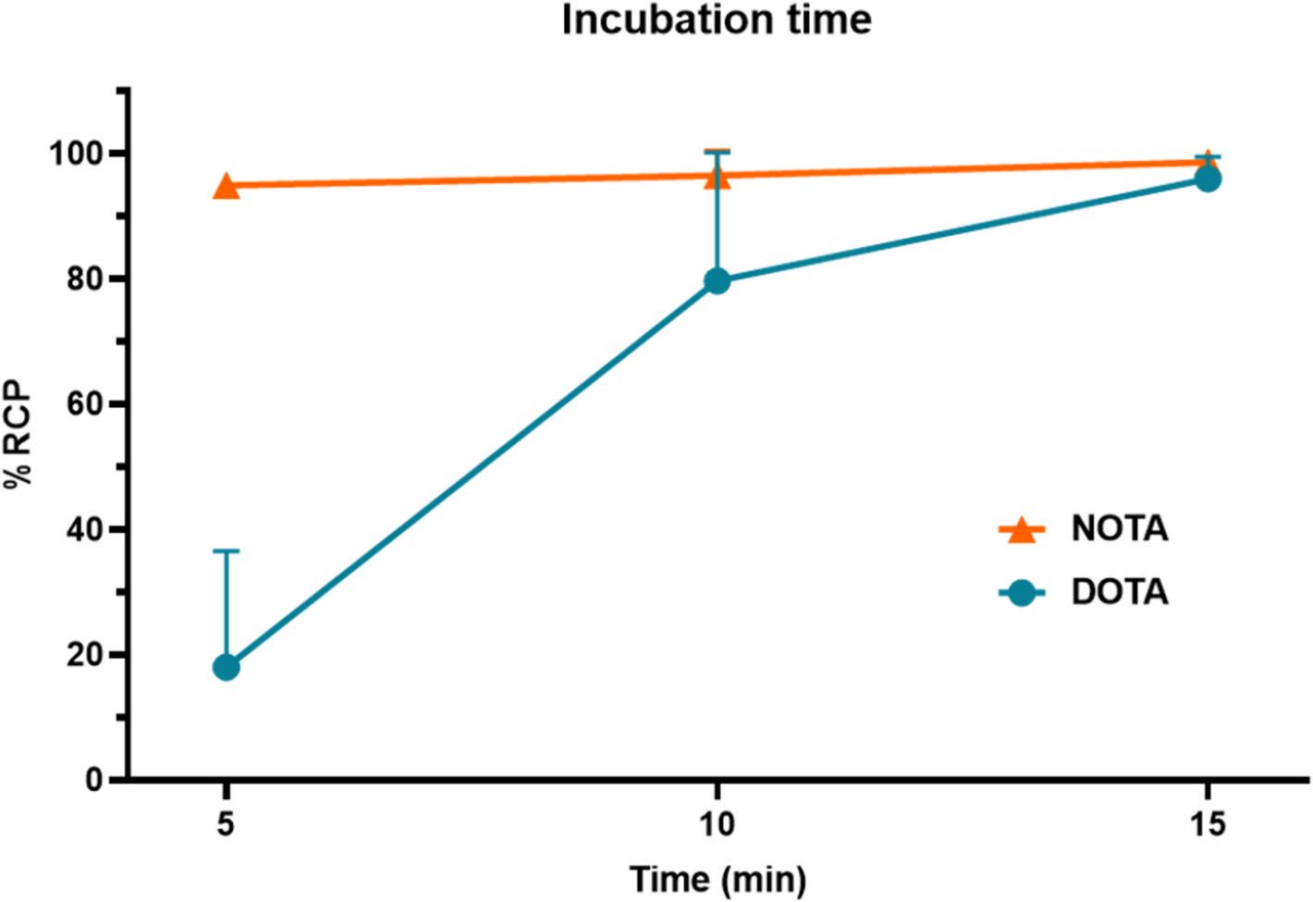
Radiochemical purity of [^68^Ga]Ga-NOTA-A1-His and [^68^Ga]Ga-DOTA-A1-His evaluated as a function of incubation time. ***P < 0.001 vs NOTA

- Reaction concentration
  The effect of A1-His concentration on the radiochemical purity (RCP) of the reactions for both [68Ga]Ga-NOTA-A1-His and [68Ga]Ga-DOTA-A1-His was investigated (Fig. 2A). The reactions were carried out at two different concentrations of A1-His: 50 µg/mL and 100 µg/mL. Radiochromatograms are available as supplemental data (Supplementary Fig. 1).
  For both chelators, the RCP was < 95% at the concentration of 50 µg/mL (85 ± 1% and 87 ± 4% for NOTA and DOTA, respectively), and the concentration of 100 µg/mL resulted in a RCP > 95% (98 ± 1% and 96 ± 3% for NOTA and DOTA, respectively). The concentration of 100 µg/mL was therefore selected for further studies.
- pH
  The radiolabeling was carried out at pH 3, 4, and 5 (Fig. 2B). For [68Ga]Ga-NOTA-A1-His, the highest RCP was observed at pH 4 where it reached 98 ± 1%. For [68Ga]Ga-DOTA-A1-His, the highest RCP was observed at pH 3. At this pH, the RCP was of 96 ± 2% and was significantly higher than that of [68Ga]Ga-NOTA-A1-His (*p* < 0.001).
- Incubation time
  The effect of incubation time on the radiochemical purity (RCP) of the reactions for both NOTA and DOTA was evaluated at three different incubation times: 5, 10, and 15 min (Fig. 3). [68Ga]Ga-NOTA-A1-His RCP was of 95 ± 2%, 97 ± 4% and 99 ± 1% at 5, 10 and 15 min, respectively. In comparison, the initial [68Ga]Ga-DOTA-A1-His RCP, determined at 5 min, was of 18 ± 19%. It then increased overtime and was > 95% at 15 min (96 ± 3%).

### Radiolabeling stability and blood distribution

**Fig. 4:**
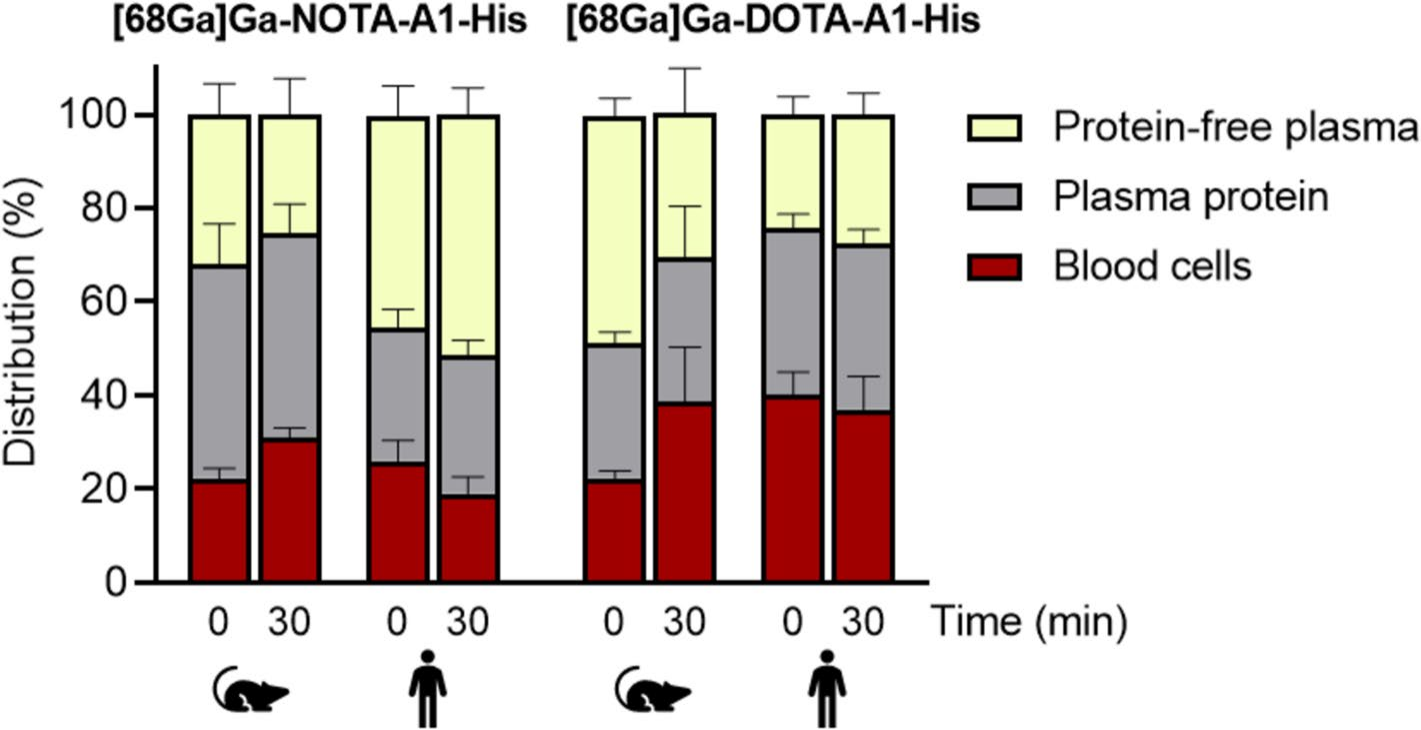
[^68^Ga]Ga-NOTA-A1-His and [^68^Ga]Ga-DOTA-A1-His distribution determined in vitro in whole human and murine blood

After identifying the best parameters for the radiolabeling, the stabilities were studied in the radiolabeling medium as well as in murine and human blood. In the radiolabeling medium, at 4 h the RCP of [68Ga]Ga-NOTA-A1-His and [68Ga]Ga-DOTA-A1-His was 98 ± 1 and 96 ± 2%, respectively. Stability in whole blood was evaluated following blood cell and large proteins removal. We took advantage of this method to also evaluate the distribution of the sdAbs between the different fractions. In vivo, this repartition could impact the availability of the sdAb for their target. Due to the very fast blood clearance of sdAbs, we focused on the early time points of 0 and 30 min when their binding occurs (Fig. 4). Blood distribution at later time point is available as supplemental data (Supplementary Fig. 2). The majority of [68Ga]Ga-NOTA-A1-His and [68Ga]Ga-DOTA-A1-His were found to be located in the plasma, either in the protein or the free fraction, both in murine and human blood. The highest activity on the blood cell fraction was observed for [68Ga]Ga-DOTA-A1-His in human blood where it was of 40 ± 5% and 37 ± 7% at 0 and 30 min, respectively (*versus* 26 ± 4 and 19 ± 4% at similar time points for [68Ga]Ga-NOTA-A1-His). In murine blood, [68Ga]Ga-NOTA-A1-His and [68Ga]Ga-DOTA-A1 distributions were found to be comparable.

**Fig. 5:**
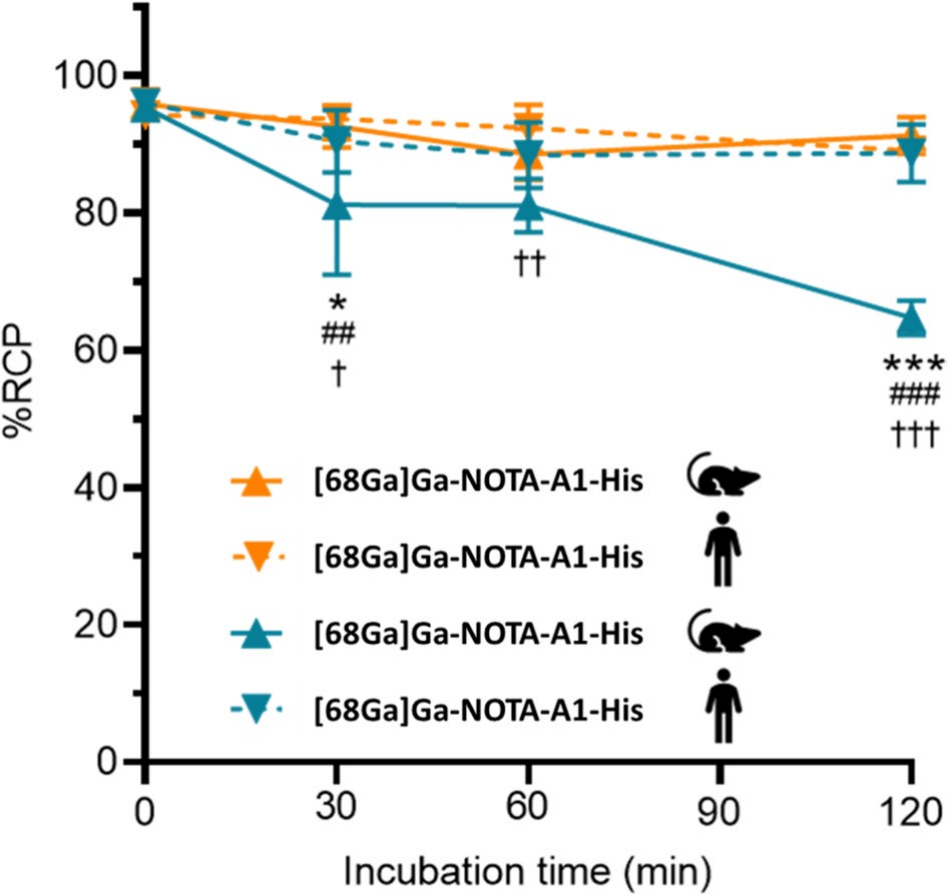
Stability of [^68^Ga]Ga-NOTA-A1-His and [^68^Ga]Ga-DOTA-A1-His in human and mouse blood. *vs human blood; # *vs* [^68^Ga]Ga-NOTA-A1-His and †*vs* [^68^Ga]Ga-NOTA-A1-His

The stability was then evaluated in the protein-free plasma fraction (Fig. 5). Elevate stabilities were observed for [68Ga]Ga-NOTA-A1-His in murine and human blood, with RCP of 91 ± 3 and 89 ± 2% at 120 min, respectively. A significant reduction in the stability was found for [68Ga]Ga-DOTA-A1 in murine blood, with RCP decreased from 95 ± 1 to 81 ± 10 at 30 min, and 65 ± 3% at 120 min. This was however not observed in human blood where the stability was similar to that of [68Ga]Ga-NOTA-A1 and was still of 89 ± 4% at 120 min.

### Hydrophilicity

Hydrophilicity’s of the two radiotracers were compared using octanol-PBS partition coefficient (logP). The LogP of [68Ga]Ga-DOTA-A1-His was determined to be -1.92 ± 0.23, while it was of − 2.24 ± 0.32 for [68Ga]Ga-NOTA-A1-His, indicating that both are hydrophilic.

### In vivo biodistribution

**Fig. 6:**
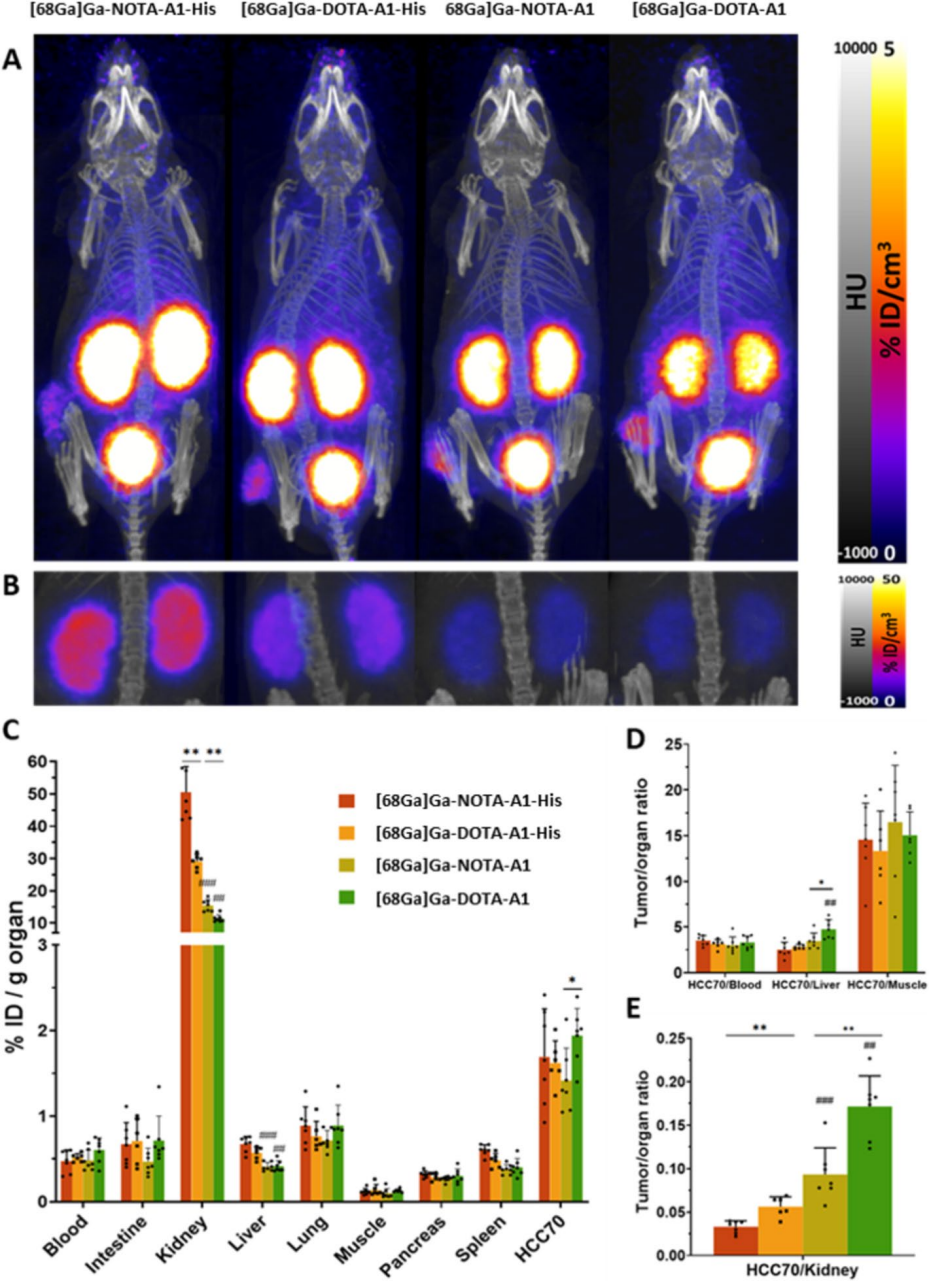
Biodistribution of [^68^Ga]Ga-NOTA-A1-His, [^68^Ga]Ga-DOTA-A1-His, [^68^Ga]Ga-DOTA-A1 and [^68^Ga]Ga-DOTA-A1 in Athymic Nude mice bearing HCC70 xenografts (n = 6–7) determined by PET/CT imaging (**A**–**B**) and ex vivo gamma well counting (**C**–**E**). **A–B:** Representative maximum intensity projection of PET/CT images acquired at 1 h after intravenous injection, using whole body field of view and a 0–5%ID/cm^3^ min–max (**A**) or a field of view focused on the kidney and a 0–50%ID/cm^3^ min–max (**B**). Biodistribution was evaluated by gamma well counting in major organs and tumor (**C**). Tumor-to-blood, tumor-to-liver, tumor-to-muscle and tumor-to-/kidney ratios were also determined (**D**–**E**). *: sdAbs [^68^Ga]Ga-DOTA labeled, significantly different from their NOTA version (*p < 0.05; **p < 0.01). ^**#**^: sdAbs without tag-his, significantly different from their tag-his version (^**##**^*p* < 0.01; ^**###**^*p* < 0.001)

By PET/CT imaging, HCC70 tumors were visible using both [68Ga]Ga-DOTA-A1-His and [68Ga]Ga-NOTA-A1-His. The signal was low in the other organs, to the exception of the kidneys and bladder. Interestingly, the kidney retention of [68Ga]Ga-NOTA-A1-His appeared to be higher than that of [68Ga]Ga-DOTA-A1-His (Fig. 6A–B). The quantification of the biodistribution by gamma well counting was in accordance with these observations (Fig. 6C–E). Indeed, the kidney retention of [68Ga]Ga-DOTA-A1-His was found to be 40% lower than [68Ga]Ga-NOTA-A1-His (29.10 ± 2.24 *vs* 50.42 ± 8.02%ID/g, *p* < 0.01). The two sdAbs without His-tag showed lower kidney retention as expected. Interestingly, the kidney retention of [68Ga]Ga-DOTA-A1 was also found to be 30% lower compared to [68Ga]Ga-NOTA-A1 (11.44 ± 1.16 *vs* 15.48 ± 1.81%ID/g, *p* < 0.01). In the other investigated organs, no major differences were observed between the two radiotracers (Supplementary Tables 2 and 3) and the activities were < 1%ID/g to the exception of the tumor where similar uptakes were found for both radiotracers. As a consequence, the tumor to kidney ratios were significantly higher using DOTA (*p* < 0.01 *vs* NOTA).

## Discussion

Both bifunctional chelating agents, NOTA and DOTA, are widely used for complexing with Gallium, each of them owning advantages and disadvantages depending on the application. NOTA forms a highly stable complex with Ga-68 (log stability constant of 30.98) (Xavier and Vaneycken 2013) and remains stable in the presence of serum cations such as Ca^2+^, Mg^2+^, and Zn^2+^. The complexation occurs with rapid kinetics, often at room temperature, making it highly suitable for labeling thermosensitive molecules like proteins. However, its structure with six coordination bonds limits its use in theranostics with radioisotopes such as Lu-177, Tb-161, or Ac-225. On the other hand, DOTA is a widely used chelating agent for a broad range of radioisotopes and is the chelating agent of choice for theranostic studies. Its drawback remains its slow kinetics, which restricts its use with thermosensitive molecules.

In this study we explored whether using these different chelators could affect the in vivo efficiency of our A1-His radioligand. To achieve this, we first established all the parameters for the conjugation and radiolabeling.

Several studies have already been conducted to compare the effects of different chelators (Krutzek et al. 2024) or more precisely NOTA and DOTA chelators in in vivo studies to complex ^68^ Ga (Dam et al. 2022; Guleria et al. 2018; Xia et al. 2020), ^64^Cu (Lee et al. 2023; Zhang et al. 2011) or ^55^Co (Mitran et al. 2019). The sdAb A1-His appears to be a good candidate to compare NOTA and DOTA using bispecific chelators and coupling on lysine residues. Indeed, the thermal stability of sdAbs is elevated in comparison to other antibody fragments (Nanobodies 2013; Bekker et al. 2019). In addition, one key challenge when conjugating a sdAb on lysine residues is avoiding potential interference with its antigen-binding specificity (Bannas et al. 2017), but there are no lysine residues in the complementarity-determining regions (CDRs) of A1-His (N’Guessan et al. 2025).

In this study, given the number of lysines present per nanobody (i.e. 4), our initial goal for conjugation was to establish conditions that would first decrease unconjugated sdAb, thereby increasing the molar activity during Ga-labeling, and second, increase the conjugation rate of the chelator per molecule. By applying the same conditions to both chelators, the initial rate of 0.4 per molecule was identical. Subsequently, by adjusting the excess of chelator and the pH, the first objective was achieved for both, with negligible levels of unconjugated sdAb under using a fifty-molar excess of chelator at pH 9. After conjugation, the sdAb exhibited elevated stability when concentrated at 1 mg/mL and stored at − 80 °C.

Optimization of radiolabeling conditions was then performed. The evaluated parameters included concentration, pH, incubation time, and molar activity. The optimal radiolabeling parameters for [68Ga]Ga-NOTA-A1-His and [68Ga]Ga-DOTA-A1-His differed regarding the pH, reflecting the structural and chemical differences between these two chelators. Studies have shown that NOTA achieves optimal radiolabeling yields at a slightly acidic pH of 4, while DOTA requires more acidic conditions, with a pH of 3, to achieve maximum radiochemical purity (Jeong and Kim 2009). This difference is attributed to the more rigid, macrocyclic structure of DOTA, which necessitates a lower pH to facilitate ^68^Ga complexation. In contrast, NOTA's slightly higher optimal pH for radiolabeling (around 4) suggests that its more flexible structure allows ^68^Ga binding under milder acidic conditions. Such distinctions underscore the need to carefully adjust pH for each chelator to maximize labeling efficiency and ensure high radiochemical purity, especially in the context of clinical applications where consistency and stability of radiotracers are critical. Consequently, selecting the appropriate pH, based on chelator properties, is essential for optimizing ^68^Ga labeling, with implications for both in vitro and in vivo performance of the resulting radiotracers.

NOTA achieves high purity at RT rapidly and consistently within 5 min, while DOTA requires a longer incubation time of 15 min and heating at 60 °C to reach comparable levels of purity. Although the NOTA‑conjugated samples looked more homogeneous across incubation times, the differences were not statistically significant. This apparent uniformity still favors reproducibility. By contrast, DOTA labeling, normally performed above 80 °C, was carried out at 60 °C to protect the sdAb. The incubation time was therefore prolonged relative to standard protocols, which may have contributed to the greater variability observed.

In the radiolabeling medium [68Ga]Ga-NOTA-A1-His and [68Ga]Ga-DOTA-A1-His demonstrated good stability up to 4 h post-incubation. In vitro in human and murine blood, the majority of [68Ga]Ga-DOTA-A1-His and [68Ga]Ga-NOTA-A1-His was found in the plasma fractions. However, [68Ga]Ga-DOTA-A1-His activity located within the blood cells in human blood was higher than that of [68Ga]Ga-NOTA-A1-His, suggesting that the bioavailability of the two radiotracers might differ following intravenous injection. Furthermore, [68Ga]Ga-DOTA-A1-His stability in murine blood was found to be lower than that of [68Ga]Ga-NOTA-A1-His or than that observed in human blood, as early as at 30 min. Those results are similar to those of the literature, such as in the comparative evaluation of [68Ga]Ga-labeled TATEs (Xia et al. 2020). This could impact its biodistribution and lower the amount of specific binding to its target. However, no such difference in tumor uptake was observed in vivo in mice bearing HCC70 tumors. This might be attributed to the fact that the blood kinetic of sdAbs is rapid and that most of the specific binding occurs in the first minutes when the RCP is elevated.

PET is a powerful non-invasive imaging modality that offers whole-body visualization, playing a critical role in oncology. Due to its elevated sensitivity and resolution, PET imaging is usually favored to SPECT when developing new radiopharmaceuticals to phenotype the tumors, monitor the disease progression including metastasis, and evaluate the efficacy of treatments (Crișan et al. 2022). In vivo in mice bearing HCC70 tumors, the biodistribution profiles of [68Ga]Ga-NOTA-A1-His and [68Ga]Ga-DOTA-A1-His were remarkably similar, demonstrating that the choice of the chelator does not affect the specific targeting of mesothelin-expressing tumors. Moreover, low bone activity confirmed the stability of the radiolabeling. However, a significant difference was observed in the renal uptake. The DOTA-conjugated sdAbs exhibited a significantly lower kidney uptake compared to the NOTA-conjugated. This reduction in renal accumulation is advantageous for imaging applications, as it enhances tumor-to-kidney contrast and even more critical for therapeutic application as it reduces the radiation exposure to the kidneys, which is a critical consideration in radiopharmaceutical development. This lower renal uptake may be attributed to the type and number of chelators attached to the sdAb molecule. During the conjugation studies, the DOTA chelator achieved a higher chelator-to-sdAb ratio (1.8) compared to NOTA (1.3). Previous research has suggested that the degree of chelator conjugation can influence the pharmacokinetics and biodistribution of sdAb-based radiotracers (Baudhuin et al. 2021). A higher chelator density might affect the overall charge, hydrophilicity, and protein binding properties of the sdAb, potentially reducing renal reabsorption and facilitating faster renal clearance. Moreover, the net charges of the conjugates could also have an impact on their in vivo kinetics and further studies will be conducted to investigate this parameter.

From a clinical translation perspective, the use of DOTA as a chelator presents several advantages. DOTA is capable of forming stable complexes not only with Gallium-68 for PET imaging but also with therapeutic radionuclides such as Lutetium-177 and Terbium-161. This versatility allows for the development of a single sdAb-based molecule that can be utilized for both diagnostic imaging and radionuclide therapy, simplifying the production process, regulatory and toxicity evaluations. The ability to use one molecule for theranostic applications aligns with the growing interest in personalized medicine, where treatments are tailored to the specific characteristics of an individual's disease. However, the practical aspects of radiolabeling also need to be considered. The NOTA chelator offers the significant advantage of rapid and efficient radiolabeling with ^68^Ga at room temperature, which is highly beneficial for clinical routine. In contrast, DOTA requires higher temperatures (up to 60 °C in our study) and incubation time a little longer than NOTA to achieve optimal radiochemical purity. This duration is however compatible with routine clinical practice, and is much more rapid than the one previously required using tricarbonyl for ^99m^Tc labeling (90 min, at 60 °C) (Montemagno et al. 2018).

## Conclusions

This study demonstrated that both DOTA and NOTA chelators can be effectively conjugated to the anti-mesothelin sdAb A1-His and radiolabeled with gallium-68. While [68Ga]Ga-NOTA-A1-His allows rapid radiolabeling at room temperature, facilitating clinical routines, [68Ga]Ga-DOTA-A1-His showed significantly lower kidney uptake and have the ability to chelate therapeutic radionuclides like lutetium-177. Moreover, his-tag removal efficiently reduced kidney retention. The sdAb [68Ga]Ga-DOTA-A1 is therefore a suitable candidate for theranostic applications.

## Supplementary Information

Additional file 1.

## Data Availability

The datasets generated and analyzed during the current study are available from the corresponding author on reasonable request.

## Abbreviations

%ID/g: Percent of injected dose per gram
^177^Lu: Lutétium-177
^68^Ga: Gallium-68
^161^Tb: Terbium-161
A1-His: Anti-mesothelin sdAb A1 with a hexahistidine tag
CT: Computed tomography
DOTA: 1,4,7,10-tetraazacyclododecane-1,4,7,10-tetraacetic acid
HER2: Human epidermal growth factor receptor 2
HPLC: High-pressure liquid chromatography
IMAC: Immobilized metal affinity chromatography
MALDI-TOF: Matrix assisted laser desorption ionization Time of flight
MMR: Macrophage mannose receptor
MSLN: Mesothelin
NOTA: 2,2′,2″-(1,4,7-triazacyclononane-1,4,7-triyl)triacetic acid
p-SCN-Bn-DOTA: p-Isothiocyanatobenzyl derivative of DOTA
p-SCN-Bn-NOTA: p-Isothiocyanatobenzyl derivative of NOTA
PBS: Phosphate buffer saline
PET: Positron emission tomography
RCP: Radiochemical purities
SD: Standard deviation
sdAb: Single domain antibody
SEC: Size exclusion chromatography
SPECT: Single photon emission computed tomography
SPR: Surface plasmon resonance
TNBC: Triple negative breast cancer
VHH: Variable regions of the heavy chain of heavy-chain-only antibodies
