# Supplementary material for: Side by side comparison of NOTA and DOTA for conjugation efficiency, gallium-68 labeling, and in vivo biodistribution of anti-mesothelin sdAb A1-His
Source: EJNMMI Radiopharm Chem. 2025 Aug 20;10:54. doi: 10.1186/s41181-025-00380-5 (PMC12367608; doi:10.1186/s41181-025-00380-5)
Supplement: Supplementary file 1 — Additional file 1. [file 41181_2025_380_MOESM1_ESM.docx]

***Side by side comparison of NOTA and DOTA for conjugation efficiency, gallium-68 labeling, and in vivo biodistribution of anti-mesothelin sdAb A1-His***

***Supplementary***

**Author names and affiliations**

*Émilien N’guessan^1^, Sandrine Bacot^1^, Florian Raes^1^, Julien Leenhardt^1, 2^, Thibault Guenard^1^, Laurent Dumas^1^, Catherine Ghezzi^1^, Daniel Fagret^1^, Charlotte Lombardi ^1, 3^, Alexis Broisat^1^, Mitra Ahmadi^1^
^1^ Univ. Grenoble Alpes, INSERM U1039, LRB, Grenoble, France*

*^2^ Univ. Grenoble Alpes, CHU Grenoble Alpes, Department of Nuclear Medicine, Grenoble, France*

*^3^ Univ. Grenoble Alpes, CNRS U5525, TIMC-TREE, La Tronche, France*

Corresponding Author: alexis.broisat@inserm.fr

***
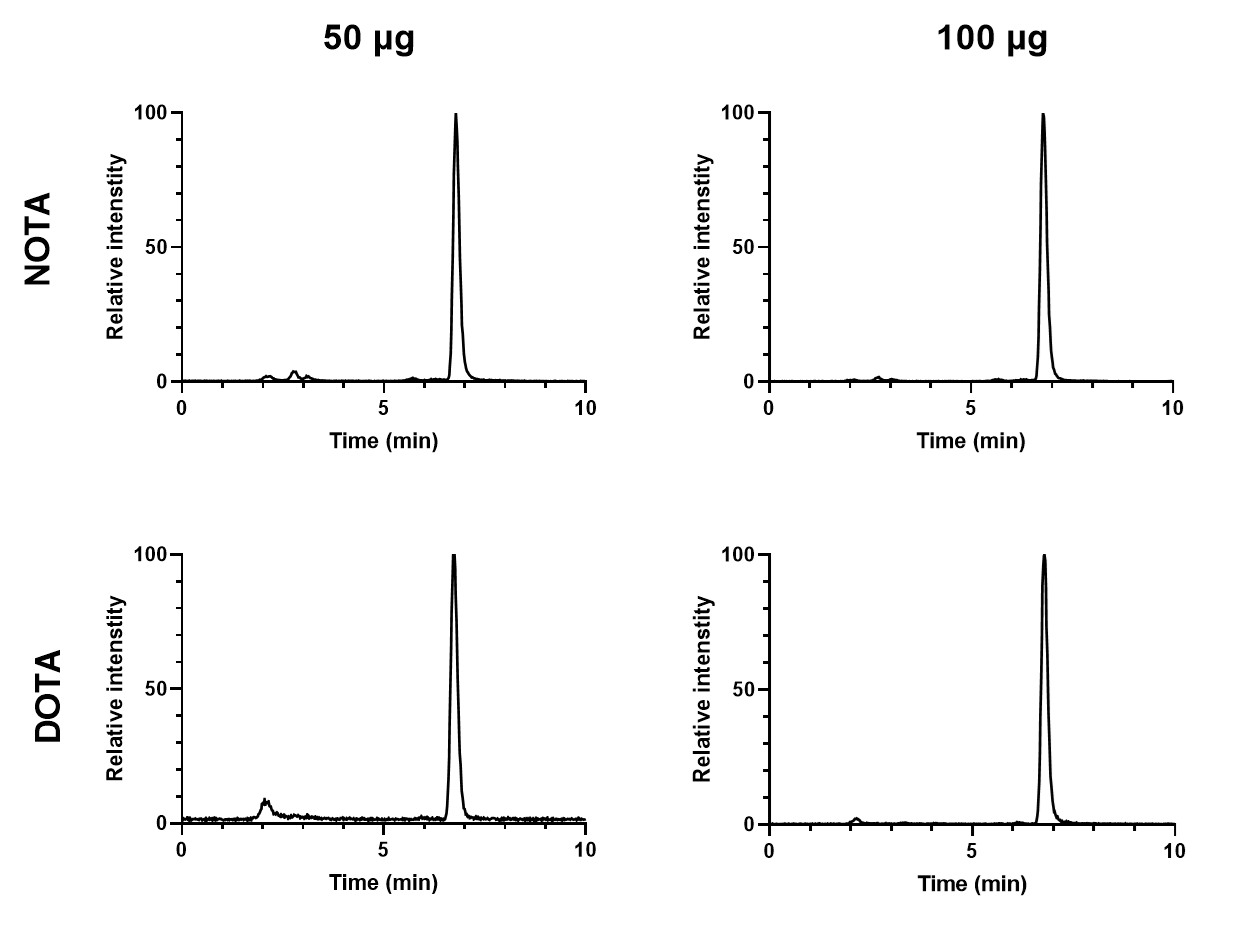
***

**Figure 1:** Radiochromatograms showing radiochemical purity of [68Ga]Ga-NOTA-A1-His and [68Ga]Ga-DOTA-A1-His immediately after radiolabeling as a function of A1-His concentration.

******

**Figure 2:** [68Ga]Ga-NOTA-A1-His and [68Ga]Ga-DOTA-A1-His distribution determined *in vitro* in whole human and murine blood up to 120 minutes

**
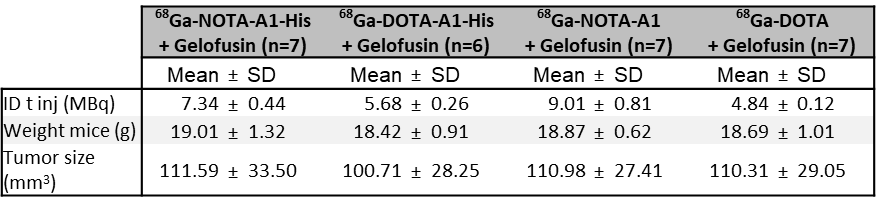
**

**Table 1.** Details of the groups of mice injected in the *in vivo* biodistribution comparisons of the sdAb [68Ga]Ga-NOTA-A1-His and [68Ga]Ga-DOTA-A1-His, and their version with no His-tag. Expressed as mean ± SD (standard deviation); (n=6-7)


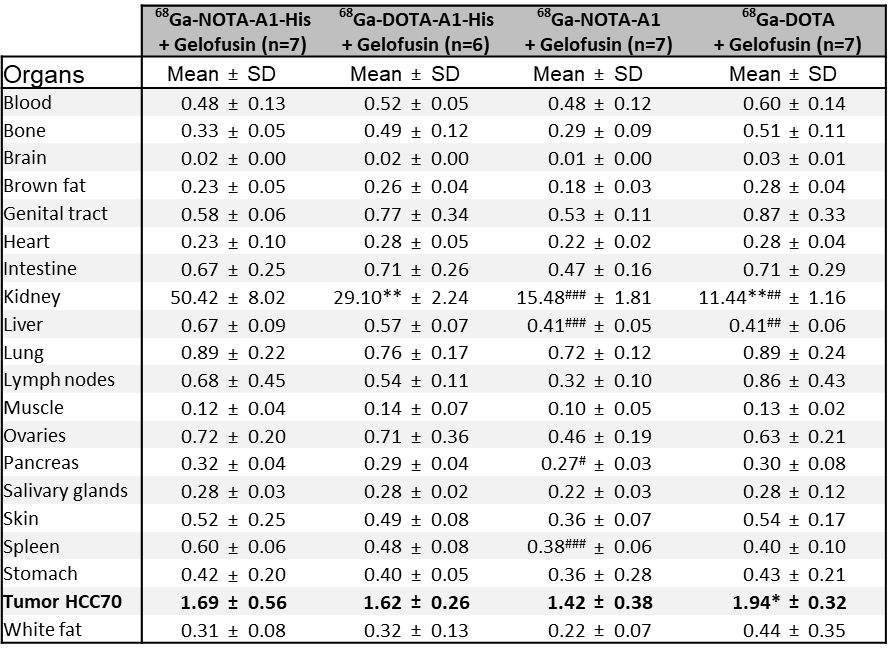
**Table 2.** *Ex vivo* biodistribution data 2 hours after intravenous injection of the sdAb [68Ga]Ga-NOTA-A1-His and [68Ga]Ga-DOTA-A1-His, and their version with no His-tag, in Athymic Nude mice bearing HCC70 xenografts, expressed as mean of % ID/g tissue ± SD (standard deviation); (n=6-7) ***:** sdAbs [68Ga]Ga-DOTA labelled, significantly different from their NOTA version (*=p < 0.05 ; **= p < 0.01). **^#^:** sdAbs without His-tag, significantly different from their tag-his version (**^##^**= p < 0.01 ; **^###^**= p < 0.001).


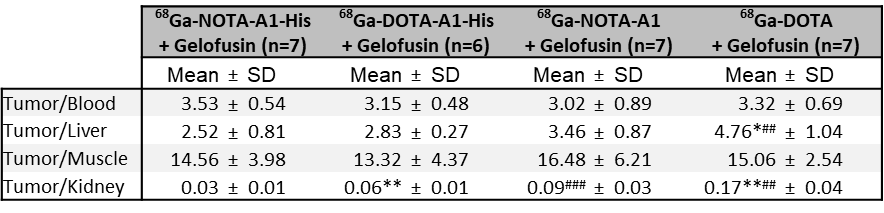


**Table 3.** Tumor-to-organ ratios of %ID/g values 2 hours after intravenous injection of the sdAb [68Ga]Ga-NOTA-A1-His and [68Ga]Ga-DOTA-A1-His, and their version with no His-tag, in Athymic Nude mice bearing HCC70 xenografts. Expressed as mean of % ID/g tissue ± SD (standard deviation); (n=6-7) ***:** sdAbs [68Ga]Ga-DOTA labelled, significantly different from their NOTA version (*=p < 0.05 ; **= p < 0.01). **^#^:** sdAbs without His-tag, significantly different from their tag-his version (**^##^**= p < 0.01 ; **^###^**= p < 0.001).
